# Dr. Lawson

**Published:** 1861-07

**Authors:** 


### NECROLOGICAL NOTICES.

DR. LAWSON, Surgeon-General of the United States Army, recently died at Old Point Comfort, at an advanced age, and after long-continued failing health. We have not seen any memoir of his life, or any of the particulars of his last illness.

